# Assessment of laboratory and daily energy expenditure estimates from consumer multi-sensor physical activity monitors

**DOI:** 10.1371/journal.pone.0171720

**Published:** 2017-02-24

**Authors:** Enhad A. Chowdhury, Max J. Western, Thomas E. Nightingale, Oliver J. Peacock, Dylan Thompson

**Affiliations:** Department for Health, University of Bath, Bath, United Kingdom; Universita degli Studi di Verona, ITALY

## Abstract

Wearable physical activity monitors are growing in popularity and provide the opportunity for large numbers of the public to self-monitor physical activity behaviours. The latest generation of these devices feature multiple sensors, ostensibly similar or even superior to advanced research instruments. However, little is known about the accuracy of their energy expenditure estimates. Here, we assessed their performance against criterion measurements in both controlled laboratory conditions (simulated activities of daily living and structured exercise) and over a 24 hour period in free-living conditions. Thirty men (n = 15) and women (n = 15) wore three multi-sensor consumer monitors (Microsoft Band, Apple Watch and Fitbit Charge HR), an accelerometry-only device as a comparison (Jawbone UP24) and validated research-grade multi-sensor devices (BodyMedia Core and individually calibrated Actiheart^™^). During discrete laboratory activities when compared against indirect calorimetry, the Apple Watch performed similarly to criterion measures. The Fitbit Charge HR was less consistent at measurement of discrete activities, but produced similar free-living estimates to the Apple Watch. Both these devices underestimated free-living energy expenditure (-394 kcal/d and -405 kcal/d, respectively; P<0.01). The multi-sensor Microsoft Band and accelerometry-only Jawbone UP24 devices underestimated most laboratory activities and substantially underestimated free-living expenditure (-1128 kcal/d and -998 kcal/d, respectively; P<0.01). None of the consumer devices were deemed equivalent to the reference method for daily energy expenditure. For all devices, there was a tendency for negative bias with greater daily energy expenditure. No consumer monitors performed as well as the research-grade devices although in some (but not all) cases, estimates were close to criterion measurements. Thus, whilst industry-led innovation has improved the accuracy of consumer monitors, these devices are not yet equivalent to the best research-grade devices or indeed equivalent to each other. We propose independent quality standards and/or accuracy ratings for consumer devices are required.

## Introduction

There has been an explosion in the availability and popularity of wearable devices that track physical activity. This sector has grown by 150% year-on-year [1], with sales of fitness trackers, sports watches and smartwatches predicted to be 116 million in 2016 [2]. Fitbit is an established provider but, recently, some of the biggest technology companies in the world (including Apple) have also entered the wearable technology sector.

Wearable devices may be effective facilitators of behaviour change, particularly because of the opportunities arising for provision of instantaneous feedback [3] and because personalised physical activity data is motivating [4]. Therefore, as these wearable devices become more commonplace, there is the potential to harness these technologies to engage patients in the self-management of their care as well as provide lifestyle information to health care providers [5]. However, despite their enormous potential, several authors have highlighted the need for studies establishing the accuracy of these instruments [6–8].

Whilst early commercially-available wearable trackers relied on movement sensors alone (e.g., accelerometers), more recent models integrate other physiological outputs in recognition of how this improves estimates of energy expenditure [9,10]. This step-change in consumer monitor sophistication has resulted in the latest generation of consumer devices sharing similar technology as established multi-sensor devices used in research studies. There have been various assessments of older generations of consumer monitors [11–18], and a recent laboratory investigation has suggested that both the Apple Watch and Fitbit Charge HR underestimate energy expenditure during a combined 1 hour protocol involving rest, walking, running and cycling [19]. However, there has been no examination of other activities of daily living or daily energy expenditure estimates of the next generation of sophisticated multi-sensor devices including those large technology companies such as Apple.

The present study therefore examined energy expenditure estimates from these multisensor consumer technologies for a range of activities in a controlled laboratory setting and during normal free-living conditions.

## Methods

Individuals participated in two main experimental elements. During both aspects, participants wore activity trackers from a range of manufacturers and different price points as well as two research-grade multi-sensor devices. These research devices (BodyMedia Core and Actiheart^™^) have been extensively validated and shown to accurately predict energy expenditure [11,20–28]. A laboratory validation was conducted where the devices were compared to indirect calorimetry as a criterion measure. Additionally, individuals wore these devices for a calendar day with the validated Actiheart^™^ research-grade device used as a reference method for daily energy expenditure. This experimental protocol was approved by the institutional review board of the University of Bath, Department for Health (EP 14/15 253) and conducted in accordance with the principles of the Declaration of Helsinki [29].

### Participants

Thirty men (n = 15) and women (n = 15) took part in this study (mean ± SD; Age: 27 ± 6 y, height: 1.72 ± 0.10 m, body mass: 69.7 ± 13.2 kg, BMI: 23.4 ± 2.5 kg/m^2^). Participants were eligible to undertake this study if they were aged 18–50 years, free from illness or injury restricting physical function and able to undertake moderate intensity exercise. All individuals provided written informed consent and were screened for eligibility to undertake exercise by way of PAR-Q prior to commencement of testing.

### Criterion indirect calorimetry

A portable metabolic system (COSMED K4b^2^, Rome, Italy) was used during the laboratory validation element of the study, with data collected continuously during activity protocols. This device has been shown to be both reliable and valid [30,31], and has also been used previously to determine criterion energy expenditure [28,32–34]. The unit was calibrated prior to each session according to manufacturer’s instructions. Continuous data from the device was filtered into 1- minute epochs, with data for each specific activity then compared on a like-for-like basis with the commercial devices.

### Consumer physical activity monitors

During laboratory and free-living testing participants wore four (two on each wrist) consumer devices appropriate for their size, with balancing of left and right arm wear for each device. Devices were worn according to manufacturers’ instructions and configured using the associated app on a bluetooth linked mobile phone for the individual’s personal details (height, body mass, age/date of birth, sex) before testing. As these consumer devices do not provide minute-by-minute data, during laboratory testing, energy expenditure estimates were obtained immediately before and after each discrete activity with the difference calculated to represent the energy expenditure of each activity. All devices were connected to associated apps running on Apple iPhone models 5 or later, running iOS 8.

#### Apple Watch

Released in April 2015, this is the first smart watch produced by Apple Inc (Cupertino, California, USA). The device includes a triaxial accelerometer as well as heart rate (HR) measurement using photoplethysmography at the wrist. The “workout” setting on the device allows selection of different activity types, which during outdoor activities can also use a paired iPhone’s GPS. During “workout” mode the HR recording function is activated continuously. When not in “workout” mode heart rate recordings are obtained every 10 minutes. The Apple Watch provides some physical activity outputs on the device itself and also fuller information (e.g. total energy expenditure data through the associated “Activity” app). During laboratory testing, total energy expenditure estimates were obtained via the app, with the Watch OS version 1.0.1 used.

#### Microsoft Band

The first activity tracker from Microsoft Corporation (Redmond, Washington, USA) this device was released in the UK in April 2015. The Microsoft Band incorporates an optical HR sensor, a triaxial accelerometer, gyrometer, GPS, ambient light sensor, skin temperature sensor, UV sensor, capacitive sensor and galvanic skin response. The device includes various exercise modes which when activated increase the frequency of HR measurement. This device provides feedback on the device itself and through an associated app “Microsoft health” and was tested using firmware version 10.2.2818.0 and app version 1.3.10506.1. During laboratory testing, total energy expenditure estimates were obtained from the device itself.

#### Fitbit Charge HR

Released in the UK in January 2015 and produced by Fitbit Inc (San Francisco, California, USA). This company was one of the first in the activity tracking market in 2007, and is currently the market leader by volume for dedicated commercial fitness tracking devices. It includes an optical HR monitor, a triaxial accelerometer and an altimeter. The Fitbit Charge HR provides feedback on the device itself and also through the associated Fitbit app (version 2.9.1 used). During laboratory testing, total energy expenditure estimates were obtained from the device itself.

#### Jawbone UP24

Produced by Jawbone (San Francisco, California, USA) this device was the second wearable device produced by this company. This device was released in November 2013 and is representative of a typical accelerometer-only device and was included in the present study to provide a comparison with the previous generation of single sensor monitors. The device has a triaxial accelerometer. The device does not possess a screen so feedback is through the associated UP app. During laboratory testing, energy expenditure estimates were obtained via the app (version 4.4).

### Research grade devices

#### Actiheart ^™^

The Actiheart^™^ (Cambridge Neurotechnology Ltd, Papworth, UK), integrates accelerometry and HR signals. The Actiheart^™^ unit has been described in detail previously [22]. The Actiheart^™^ has previously been validated against doubly labelled water [24,26,27] and has been used as a criterion measure of free-living energy expenditure in epidemiology studies [35]. In order to individually calibrate the Actiheart^™^, the relationship between energy expenditure and HR in participants was determined at rest and during submaximal exercise at a separate visit. Individual calibration has been shown to improve energy expenditure prediction in free-living [24,26,27] and laboratory settings [23,25].

Resting metabolic rate (RMR) was measured following fifteen minutes of rest in a seated positon in accordance with minimal criteria for best practice recommendations [36], and taken as the average of fifteen minutes rest. The submaximal exercise test undertaken consisted of four 4-minute stages of incremental exercise intensity performed on a motorised treadmill (HP Cosmos Saturn 250/100r, HaB International Ltd, UK). Energy expenditure and HR were measured using the K4b^2^ and a Polar T31 HR monitor (Polar Electro Inc., NY, USA), respectively. As the output for minute-by-minute energy expenditure for the Actiheart^™^ only includes activity energy expenditure, for equivalence with the other devices during laboratory testing, the measured RMR was added. For free-living comparisons, the daily energy estimate setting within the Actiheart^™^ software added measured daily RMR and estimated dietary induced thermogenesis to the activity energy expenditure in order to obtain total energy expenditure.

#### BodyMedia Core

The BodyMedia Core armband is a research-grade device produced by BodyMedia Inc., Pittsburgh, PA. This device combines a tri-axial accelerometer with heat-related sensors (heat flux, skin temperature, near body ambient temperature) and galvanic skin response to estimate energy expenditure using proprietary algorithms. Data files from the BodyMedia Core were processed using SenseWear^®^ Pro 8.0, algorithm v5.2. Previous research has shown that BodyMedia SenseWear accurately measures energy expenditure relative to criterion measures [11,20,21] and has been used to quantify physical activity and energy expenditure in experimental trials [37,38].

### Laboratory activity protocol

Participants arrived at the laboratory having abstained from food intake, caffeine intake and exercise in the 4 hours prior to testing. Before and after each individual activity, energy expenditure estimates from the devices were obtained either from the devices themselves or the associated apps as described above. Participants undertook two activity blocks during the laboratory protocol. The first block consisted of a 24-minute protocol comprising of 4 activities of 5 minute duration conducted in the same order in all participants (seated typing on a laptop computer, simulated loading and unloading of a dishwasher, sweeping of light objects across a 3m distance and self-paced ascending and descending of one flight of stairs). During these activities, it was ensured that participants conducted activities using a relatively equal contribution from both hands.

Following a seated break of 10–15 minutes during which participants removed the portable indirect calorimeter device participants then commenced a second block of “exercise” activity lasting 64 minutes in total. Participants undertook 4 activities of 10 minutes duration completed in the same order in all participants. These activities were walking on the motorised treadmill at either 4 or 4.8 km/h, walking at the same speed with shopping bags (6kg distributed in two bags for females and 10kg distributed in 4 bags for males), cycling (at 75W for females and 100W for males) on an ergometer (Lode Excalibur Sport, Groningen, The Netherlands) and jogging on the motorised treadmill at 8.4km/h. For the cycling and jogging activities, the Apple Watch and Microsoft Band were placed into the relevant exercise settings (indoor cycle and indoor run). As the Fitbit Charge HR does not have specific exercise modes selectable, this was placed into “exercise” mode for both activities. The Jawbone UP24 did not have any specific modes for activation. All activities were followed by a 5-minute stationary standing rest period, apart from a 5-minute seated rest period following cycling. Following each rest period, there was a 1-minute transition period prior to commencing the next activity.

### Free-living data collection

Participants were shown how to use the activity trackers and informed to wear the devices at all times apart from during contact with water. They were instructed to use relevant available modes where applicable. The participants were told that there were no restrictions on their activity for the recording period and wore the devices for a minimum of 36 hours, such that one full calendar day was obtained of activity measurements. After a day of wear, these devices were collected from participants and the relevant data recorded by experimenters.

### Statistical analysis

Predicted energy expenditure data from each wearable device was compared to corresponding criterion energy expenditure data for each activity. Statistical significance was set *a priori* at α < 0.05. Analyses of agreement were conducted comparing an assigned criterion (laboratory; indirect calorimetry, free-living: individually calibrated Actiheart^™^) and predicted energy expenditure from each device using Bland and Altman plots to calculate absolute bias and 95% limits of agreement (LoA). Other comparison statistics were also calculated including mean signed error (MSE) and mean absolute error (MAE) for each activity. As it is likely the absolute error of estimation will increase with exercise intensity [39] and to allow comparison between activities, error of estimate data is presented as a percentage (absolute kcal/min is presented in Table 1). Root mean squared error and Pearson product-moment correlations relative to criterion measures for laboratory activities and free-living is also presented in supplementary materials (S1 and S2 Tables). Repeated measures ANOVA was conducted on free-living energy expenditure estimates from devices, with pairwise comparisons conducted with a Holm-Bonferonni stepwise adjustment to prevent inflation of type 1 error [40]. For further context, as proposed by other authors [11], “equivalence testing” was conducted to compare the equivalence between the consumer devices and free-living criterion measure. For devices to be considered equivalent to the criterion with 95% precision, the 90% confidence interval of the mean must fall within the proposed equivalence zone. Based upon previous work this equivalence zone was determined as ±10% of the criterion mean [11].

**Table 1:** Measured energy expenditure (± SD) of the activities undertaken.

Activity	Measured EE (kcal.min^-1^)	Microsoft Band (kcal.min^-1^)	Apple Watch (kcal.min^-1^)	Fitbit Charge HR (kcal.min^-1^)	Jawbone UP24 (kcal.min^-1^)	Bodymedia Armband (kcal.min^-1^)	Actiheart (kcal.min^-1^)	METs (calculated)
**Typing**	1.51 ± 0.39	1.56 ± 0.33	2.10 ± 0.51	1.42 ± 0.35	1.49 ± 0.33	1.72 ± 0.35	1.25 ± 0.35	1.4 ± 0.2
**Dishwasher**	3.69 ± 1.11	1.61 ± 0.34	2.40 ± 0.58	3.36 ± 1.14	1.88 ± 0.84	5.57 ± 2.36	2.96± 1.25	3.5 ± 0.8
**Sweeping**	4.03 ± 1.07	2.14 ± 0.58	2.85 ± 0.93	5.20 ± 1.42	2.67 ± 1.36	6.37 ± 2.33	3.18 ± 1.23	3.9 ± 1.0
**Stairs**	7.19 ±1.48	2.74 ± 0.82	5.43 ± 1.87	7.17 ± 2.08	5.15 ± 1.36	5.72 ± 1.32	6.04 ± 2.22	7.0 ± 1.3
**Walk**	4.12 ± 0.97	2.67 ± 0.91	4.56 ± 1.47	6.99 ± 1.63	5.08 ± 1.08	4.47 ± 1.03	4.12 ± 1.30	3.9 ± 0.7
**Loaded walk**	4.91 ± 1.28	2.41 ± 0.59	4.84 ± 1.58	7.07 ± 1.34	5.17 ± 1.06	5.55 ± 0.94	4.79 ± 1.62	4.7 ± 0.9
**Cycle**	7.44 ± 1.42	5.24 ± 2.77	7.21 ± 2.63	3.53 ± 2.00	1.30 ± 0.21	6.49 ± 1.90	5.55 ± 1.95	7.2 ± 1.3
**Run**	9.90 ± 2.01	10.32 ± 2.56	11.47 ± 2.85	10.36 ± 2.34	12.62 ± 3.14	10.61 ± 2.19	10.22 ± 2.54	9.6 ± 1.8

## Results

### Laboratory validation

**Fig 1:**
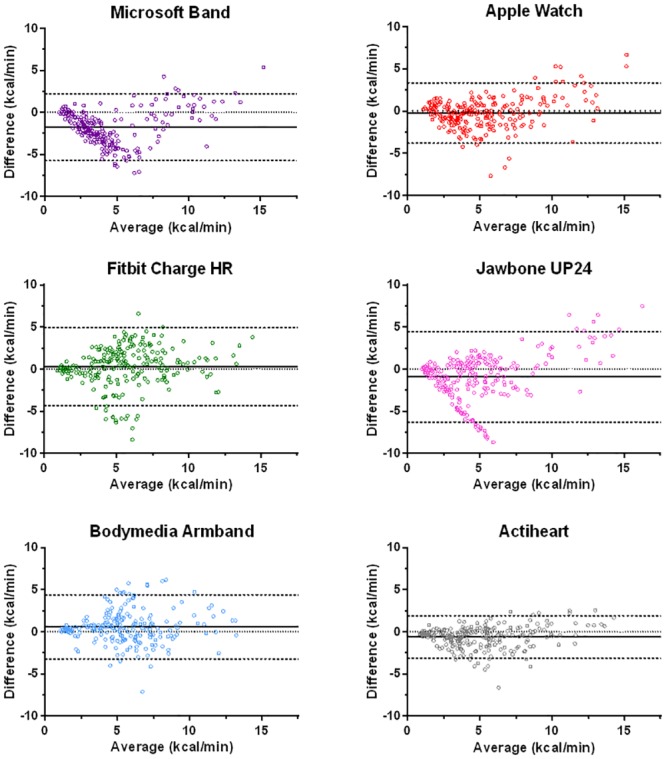
Bland-Altman plots for laboratory activities. Bland-Altman plots depicting absolute bias and 95% limits of agreement of estimated energy expenditure across a number of different activities for a range of activity trackers relative to the criterion indirect calorimetry measure. Bias represents predicted expenditure-criterion expenditure. The solid line represents mean bias, the dashed lines the upper and lower 95% limits of agreement and the dotted line represents perfect agreement.

Bland and Altman plots (Fig 1) illustrate the agreement between criterion and predicted energy expenditure for each device by displaying the mean difference and 95% LoA. For the commercially available monitors, the absolute bias ± 95% LoA values were -1.8 ± 3.9, -0.2 ± 3.4, 0.3 ± 4.6 and -0.9 ± 5.4 kcal/min for the Microsoft Band, Apple Watch, Fitbit Charge HR and Jawbone UP24, respectively. Visual inspection of the plots for the consumer devices highlights some tendency for over prediction of higher intensity activity relative to more frequent under prediction of lower intensity activities. Of the research grade wearable monitors, absolute bias ± 95% LoA values were 0.6 ± 3.8 kcal/min for the Bodymedia Armband with the smallest limits of agreement of any device observed for the Actiheart (-0.6 ± 2.5 kcal/min).

**Fig 2:**
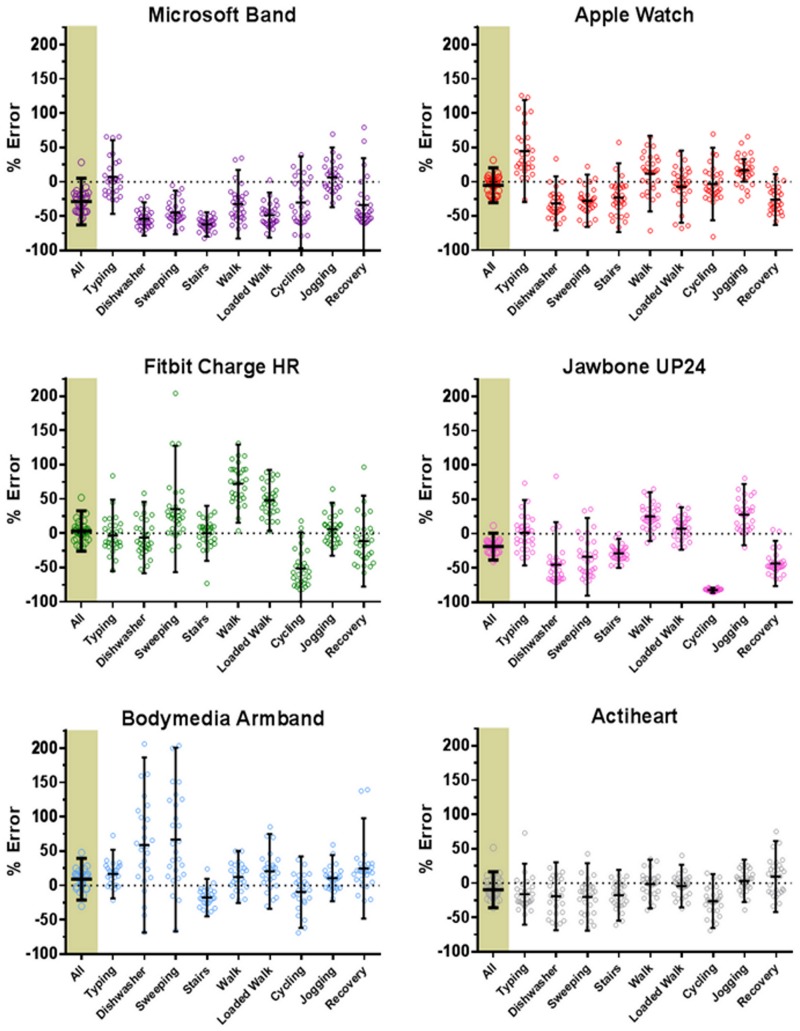
Percentage error of estimation of laboratory activities. Modified box and whisker plot depicting the percentage error of estimation relative to criterion indirect calorimetry for each activity undertaken. The highlighted ‘All’ data furthest left represents the combination of all the activities and recovery periods considered as a whole block. Recovery data represents the combination of the 5 minute stationary recovery periods after the 10 minute walk, loaded walk, cycle and jog periods. Small circles represent the individual data points, with horizontal bars representing the mean for each activity bordered by the 95% limits of agreement.

Modified box and whisker plots (Fig 2) present the overall and activity specific percentage error of estimate (± 95% LoA) for all devices, with absolute energy expenditure for each activity displayed in Table 1. Fig 2 shows variability between and within monitors for specific activities. However, looking solely at directional error can be misleading as under and over-predictions can cancel each other out [41]. Therefore we have presented mean absolute error in Table 2. Absolute errors of estimation for the activities considered separately (the mean of the absolute error of the individual activities) was lowest amongst the consumer monitoring devices for the Apple Watch (27 ± 10%) and this was not enormously different to that recorded for the Actiheart research device (20 ± 7%). This analysis is useful for understanding cumulative error but it also assumes that gross error for each type of activity is equally important (e.g., error for typing and walking), and irrespective of activity intensity, when, of course, this will not be the case outside the laboratory.

**Table 2:** Mean absolute percent error (± SD) of the activities undertaken.

Activity	Mean Absolute Percent Error (%)					
	Microsoft Band	Apple Watch	Fitbit Charge HR	Jawbone UP24	Bodymedia Armband	Actiheart
**Typing**	20 ± 19	47 ± 36	20 ± 18	18 ± 16	19 ± 15	23 ± 15
**Dishwasher**	54 ± 12	34 ± 16	23 ± 14	52 ± 18	71 ± 52	25 ± 19
**Sweeping**	45 ± 16	30 ± 16	39 ± 44	39 ± 21	75 ± 59	25 ± 20
**Stairs**	62 ± 9	29 ± 19	15 ± 14	29 ± 11	20 ± 11	20 ± 16
**Walk**	37 ± 17	24 ± 18	73 ± 29	27 ± 16	18 ± 14	13 ± 12
**Loaded walk**	49 ± 16	14 ± 13	48 ± 23	14 ± 10	27 ± 21	12 ± 11
**Cycle**	38 ± 24	20 ± 18	53 ± 23	82 ± 2	21 ± 19	28 ± 17
**Run**	18 ± 15	22 ± 16	16 ± 13	29 ± 21	15 ± 14	13 ± 10
**Mean**	40 ± 16	27 ± 19	36 ± 22	36 ± 14	33 ± 26	20 ± 15

### Free living energy expenditure

**Fig 3:**
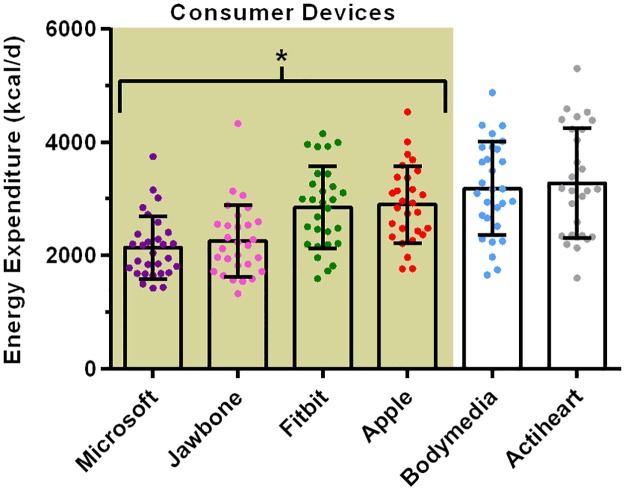
Estimated 24 hr energy expenditure from the wearable devices. Data is depicted as mean, with individual data points shown and error bars representing the SD. * Denotes that all consumer devices were significantly different p < 0.01 from the research devices. Each individual device is significantly different p < 0.01 from every other device, except for the comparisons between Microsoft *vs*. Jawbone, Fitbit *vs*. Apple and Bodymedia *vs*. Actiheart.

Data for estimated 24 hour energy expenditure is displayed in Fig 3. Pairwise comparisons indicated that all of the consumer devices produced significantly different estimates from the research grade devices (which were not different from each other). The mean absolute percent error relative to the Actiheart device was greatest in the Microsoft Band (34 ± 10%), followed by a similar magnitude of error for the Jawbone device (30 ± 11%). Both the Apple and Fitbit devices produced considerably lower error (15 ± 10% and 16 ± 8%, respectively).

**Fig 4:**
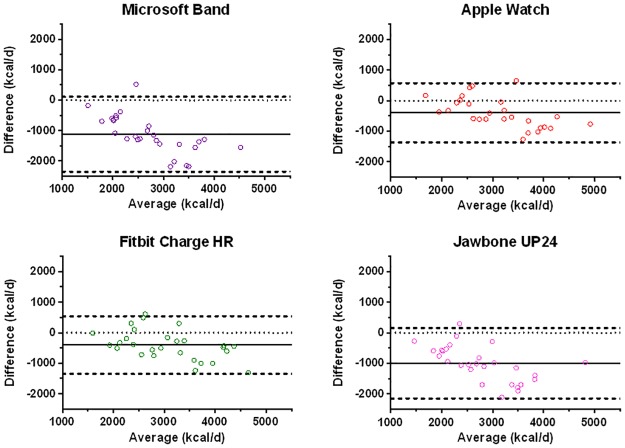
Bland-Altman plots for daily energy expenditure. Bland-Altman plots depicting bias and 95% limits of agreement of 24 hr energy expenditure for a range of commercial activity trackers relative to the criterion individually calibrated Actiheart device. Bias represents (predicted expenditure—criterion expenditure). The solid line represents absolute bias, the dashed lines the upper and lower 95% limits of agreement and the dotted line represents perfect agreement.

Bland and Altman plots (Fig 4) illustrate the agreement between the activity trackers and the criterion measure for free-living energy expenditure. Both the Fitbit Charge HR (-405 ± 944 kcal/d) and Apple Watch (-394 ± 970 kcal/d) displayed similar 95% limits of agreement. The Microsoft Band and Jawbone UP24 devices displayed much greater bias and wider limits of agreement of -1123 ± 1235 kcal/d and -998 ± 1153 kcal/d, respectively. Visual inspection of the Bland-Altman plots shows increasing negative bias with increasing daily energy expenditure for all of the consumer devices, with this effect particularly pronounced for the Microsoft Band and Jawbone UP24. For reference, the research grade Bodymedia device had similar limits of agreement to the Apple and Fitbit devices but with lower mean bias when compared to individually-calibrated Actiheart^™^ (-66 ± 965 kcal/d).

**Fig 5:**
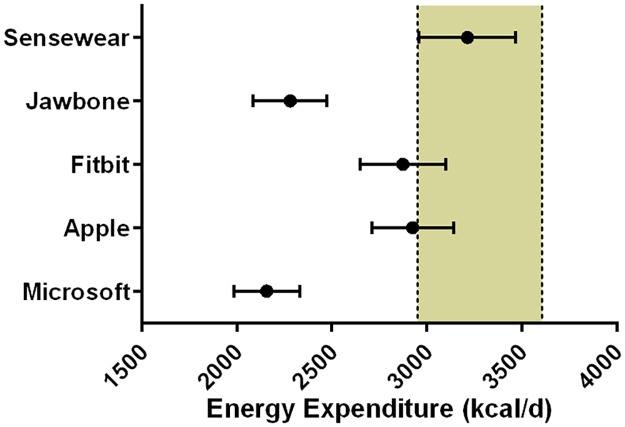
95% equivalence testing for daily energy expenditure. The shaded area bordered by dashed black lines is the proposed equivalence zone (±10% of the criterion mean). The mean of the daily energy expenditure estimates is shown for the devices, bordered by 90% confidence intervals.

For further context, as suggested by other authors, 95% equivalence testing was conducted [11]. Fig 5 demonstrates that the Sensewear device was the only monitor deemed equivalent to the Actiheart^™^ device for daily energy expenditure estimates, with none of the consumer devices lying within the proposed equivalence zone of ±10% of the criterion mean.

## Discussion

This study demonstrates that the gap between consumer and research-grade devices was only modest in some cases and industry-led technological innovation is clearly improving the accuracy of physical activity monitors that are available to consumers. However, performance was not consistently good across all devices; sometimes in spite of the inclusion of a plethora of sensors. Clinicians and the public should not assume that all devices provide accurate daily energy expenditure estimates and certainly they are not equivalent.

Across all assessments, strong overall performance was observed for the Apple Watch, which displayed the tightest limits of agreement and mean absolute error of the consumer devices in the laboratory assessments. The Apple Watch was also the closest of the consumer devices in free-living conditions to the Actiheart^™^ for assessing daily energy expenditure. In spite of its many sensors, there was consistent under prediction from the Microsoft Band both inside and outside the laboratory. Of the other devices, less consistent prediction of the energy expenditure of discrete specific activities was observed from the Fitbit Charge HR in the laboratory assessments, although the free-living daily expenditure estimates from this device were similar to the Apple Watch. Therefore, in comparison with the Apple device, it appears that despite more marked error in estimating the expenditure of some specific activities, this does not have a detrimental effect upon the total expenditure estimates from this device outside the laboratory. As discussed below, this probably reflects the contrived nature of laboratory-based assessments, and a strength of this work is that we also obtained energy expenditure estimates during free-living. This provides the most ecologically valid assessment of these monitors carrying out their designated function and showed that all of the consumer devices underestimated daily energy expenditure relative to the reference method (individually calibrated Actiheart^™^).

To our knowledge, this is the first examination of these consumer multi-sensor devices in both laboratory and free-living settings. Our results demonstrate that these sophisticated devices are yet to match the consistency of the best performing research device in measuring discrete activities. The pattern of error for the commercial devices indicated a greater degree of underestimation relative to the Actiheart^™^ device with greater daily energy expenditure. As a substantial contributor to total energy expenditure (particularly with lower levels of activity) will be RMR, which is a broadly predictable component of energy expenditure [42], the greater accuracy for individuals with lower energy expenditure is unsurprising. Assuming relative similarity of resting energy expenditure estimates between devices, this variance in total energy expenditure estimates between devices suggests that the measurement of physical activity *per se* is more variable than the estimation of total energy expenditure would suggest. Indeed, the marked underestimation with increasing energy expenditure for certain monitors highlights there are ongoing discrepancies in the ability to translate inputs from sensors into estimates of physical activity expenditure.

Our work has important implications for researchers undertaking monitor evaluations. Commonly, laboratory validations employ protocols comprising a variety of activities treated as a single block to represent “overall” monitor performance. The rationale for this approach is that devices will over predict for some activities and under predict for others, with the assumption that the most relevant outcome is the estimate derived from a sustained period of wear time [43]. Laboratory and free-living assessments both give valuable but unique information with which to assess monitor performance in different contexts. Laboratory assessments can produce data about specific activities which is useful for individuals interested in measuring a particular activity (e.g., someone taking up jogging wanting to assess energy expenditure during running). However, laboratory protocols tend to overemphasise active elements of daily living, relative to the reported 52% sedentary time from 24 hour measured activity in young men [44]. Furthermore, the specifics of any given activity protocol (including our own) is unlikely to represent the distribution and importance of various activities in normal life. Indeed, Murakami and colleagues (2016), have shown that estimates for the same devices assessed during a standardized day and free-living wear do not necessarily produce similar directions or magnitudes of error in the different settings [18]. Therefore, it is beneficial to assess prolonged wear in free-living conditions in addition to laboratory assessments of monitor performance for specific activities.

The large discrepancies in estimating daily energy expenditure between the various activity trackers illustrates that the public and practitioners need to be cautious when using these devices to guide lifestyle decisions around energy balance. The difference between the group mean from the device predicting the lowest and highest energy expenditure (Microsoft Band *versus* Apple Watch) was >750 kcal/d which is clearly a meaningful difference. This highlights that medical and other practitioners should be wary of attempting to use devices of this nature to provide guidance on energy intake or balance without prior knowledge of their accuracy, particularly as our data illustrate substantial individual variability of estimates. The applied importance of the accuracy of energy expenditure estimates for energy balance has recently been highlighted by McCaig and colleagues (2016). These authors have demonstrated that the framing of a set bout of exercise (50 kcal *vs* 265 kcal) affects subsequent intake, with higher energy expenditure information providing greater “licence to eat” [45].

There are some considerations that should be highlighted when interpreting our work. Aligned with the suggested predominant users of these devices, we have examined a relatively young, healthy and active population [7,46]. We have established “out of box” performance for these devices, it is therefore possible that some additional calibration that is available on some devices with prolonged wear (e.g. the Apple watch claims to improve accuracy of estimation with greater use) may improve accuracy. For the laboratory validation element, several of these devices were used in a specific exercise mode that reflected indoor use. As of yet, the relative importance of specific modes is unknown and this requires clarification so users know how the level of interaction with their wearable influences device accuracy. Additionally, due to the nature of the devices tested, it is not possible to conclusively establish if there is a lag in the devices picking up specific activities, so protocols involving longer activity durations may be required. Further studies should examine the performance of these devices in other target groups, using a longer period of free-living and ideally with doubly labelled water as a criterion energy expenditure measure.

We acknowledge that absolute estimation of energy expenditure may be less important to some individuals. If the purpose of a device is to self-monitor physical activity, as long as the same monitor is used consistently and it provides the same result for a given activity, absolute expenditure may be less relevant. Whilst total expenditure is the most important metric for individuals whose concern is weight, this represents only one of several health harnessing aspects of physical activity [47]. However, inaccurate energy expenditure estimation does not maximise the potential utility of these devices and ideally they should be both reliable and accurate.

Most commercial devices provide features such as activity profiling, feedback and motivational cues that may be of benefit in enhancing motivation [4] and are currently being examined in a number of intervention contexts [37,48]. From the perspective of activity profiling and feedback, we recorded metrics relating to “active time” that each consumer device provided (see S3 Table for summary). Estimates ranged from 818 ± 88 min (Apple, “Total Active Time”) to 70 ± 59 min (Fitbit, “Active Minutes”) illustrating that individuals already attempting to establish physical activity status against a plethora of physical activity benchmarks [49] may easily be confused by such variable estimates for ostensibly similar outcomes. This discrepancy for similar outcomes was also apparent for the two devices that provided a metric for resting energy expenditure (Apple: 2181 ± 414 kcal/d and Jawbone 1494 ± 230 kcal/d). Unfortunately, none of the commercial devices tested readily allowed exporting of the raw energy expenditure data for detailed interrogation that might be useful for research/health applications.

Our data shows that when it comes to activity tracking, a greater number of sensors does not automatically guarantee more accurate energy expenditure estimation. Regrettably, at present, as independent authors (and indeed the research community as a whole) we can say very little about potential reasons for the results we obtain when examining devices of this nature. Perhaps understandably, manufacturers do not release the algorithms used in estimation of energy expenditure. However, as suggested by van Hees and colleagues (2016) there are a number of steps manufacturers could take such as providing detailed specifications of sensors, or documentation of algorithms etc. that would allow greater evaluation of these devices [50]. While it is potentially optimistic to expect this degree of cooperation between manufacturers and the research community, it is imperative that the public are at least provided with information as to the validity of these devices.

So, what could be done in order to increase the utility of these devices for use by practitioners and the public? In the future, accreditation and/or regulation of these devices (as has been suggested for mHealth apps [51]) within predefined boundaries of accuracy based upon standardised testing protocols should improve the quality of devices. These independent criteria could be similar to those the National Institute of Science and Technology issue for numerous other measurement devices in the United States, or “British Standard” marks in the United Kingdom. While other authors have suggested 10% as an appropriate equivalency relative to a criterion measure [11], more work is needed to establish a consensus acceptable error boundary for devices of this nature. Alternatively, in a similar way to efficiency ratings for household devices, activity trackers could be graded to allow the end user to incorporate accuracy as a consideration when purchasing devices.

## Conclusions

Some consumer multi-sensor devices such as the Apple Watch and Fitbit Charge HR provided reasonably good estimates of energy expenditure both in the laboratory and during free-living conditions. However, this was not the case across all devices and certainly these consumer monitors do not all produce similar results (i.e., they are not equivalent). It is also clear that devices with more sensors do not necessarily produce better energy expenditure estimates than simple previous-generation accelerometry-only devices. We propose that independent quality standards should be developed to verify claims regarding energy expenditure estimation from consumer wearables or manufacturers should be required to provide accuracy ‘ratings’ at the time of going to market.

## Supporting information

S1 Table Root mean square error of activities undertaken. (DOCX)

S2 Table Pearson product-moment correlation coefficient with criterion measures. The data presented above represents the Pearson product-moment correlation coefficient (r) between all of the measured data points during the laboratory protocol for each device compared against the indirect calorimetry criterion for laboratory activities. For the 24 hour free-living period the devices are correlated with the Actiheart as the criterion measure for that element of testing.
(DOCX)

S3 Table Minutes of active time as reported by consumer devices. (DOCX)
