# Supplementary material for: Assessment of laboratory and daily energy expenditure estimates from consumer multi-sensor physical activity monitors
Source: PLoS One. 2017 Feb 24;12(2):e0171720. doi: 10.1371/journal.pone.0171720 (PMC5325221; doi:10.1371/journal.pone.0171720)
Supplement: S1 Table — (DOCX) [file pone.0171720.s001.docx]

Supplementary Table 1 – Root mean square error of activities undertaken

|  | **Root Mean Square Error (kcal)** | | | | | |
| --- | --- | --- | --- | --- | --- | --- |
| **Activity** | **Microsoft Band** | **Apple Watch** | **Fitbit Charge HR** | **Jawbone UP24** | **Bodymedia Armband** | **Actiheart** |
| **Typing** | 1.92 | 3.83 | 2.05 | 1.73 | 1.65 | 2.15 |
| **Dishwasher** | 11.57 | 8.05 | 5.15 | 11.21 | 14.55 | 6.12 |
| **Sweeping** | 10.72 | 7.34 | 9.20 | 8.92 | 16.19 | 6.92 |
| **Stairs** | 23.25 | 13.27 | 7.19 | 11.03 | 8.09 | 8.79 |
|  |  |  |  |  |  |  |
| **Walk** | 18.25 | 13.04 | 30.71 | 11.61 | 8.60 | 7.34 |
| **Loaded walk** | 27.73 | 9.86 | 23.02 | 7.94 | 13.75 | 7.74 |
| **Cycle** | 32.70 | 21.97 | 45.14 | 62.60 | 22.42 | 24.83 |
| **Run** | 20.55 | 26.41 | 17.87 | 35.15 | 17.91 | 15.26 |
|  |  |  |  |  |  |  |
| **24 hour**  **Free-Living** | 1282 | 625 | 623 | 1153 | 488 | NA |
