# Supplementary material for: Assessment of laboratory and daily energy expenditure estimates from consumer multi-sensor physical activity monitors
Source: PLoS One. 2017 Feb 24;12(2):e0171720. doi: 10.1371/journal.pone.0171720 (PMC5325221; doi:10.1371/journal.pone.0171720)
Supplement: S2 Table — The data presented above represents the Pearson product-moment correlation coefficient (r) between all of the measured data points during the laboratory protocol for each device compared against the indirect calorimetry criterion for laboratory activities. For the 24 hour free-living period the devices are correlated with the Actiheart as the criterion measure for that element of testing. (DOCX) [file pone.0171720.s002.docx]

Supplementary Table 2 – Pearson product-moment correlation coefficient with Criterion Measures

|  | **Correlation Coefficient with Criterion Measures** | | | | | |
| --- | --- | --- | --- | --- | --- | --- |
|  | **Microsoft Band** | **Apple Watch** | **Fitbit Charge HR** | **Jawbone UP24** | **Bodymedia Armband** | **Actiheart** |
| **Laboratory Activities** | 0.879 | 0.935 | 0.825 | 0.800 | 0.921 | 0.945 |
| **24 hours**  **Free-living** | 0.787 | 0.873 | 0.875 | 0.810 | 0.861 | NA |

The data presented above represents the Pearson product-moment correlation coefficient (r) between all of the measured data points during the laboratory protocol for each device compared against the indirect calorimetry criterion for laboratory activities. For the 24 hour free-living period the devices are correlated with the Actiheart as the criterion measure for that element of testing.
