# Supplementary material for: Assessment of laboratory and daily energy expenditure estimates from consumer multi-sensor physical activity monitors
Source: PLoS One. 2017 Feb 24;12(2):e0171720. doi: 10.1371/journal.pone.0171720 (PMC5325221; doi:10.1371/journal.pone.0171720)
Supplement: S3 Table — (DOCX) [file pone.0171720.s003.docx]

Supplementary Table 3 – Minutes of active time as reported by consumer devices (± SD)

| **Microsoft Band**  **(Active Time)** | **Apple Watch**  **(Exercise Minutes)** | **Apple Watch**  **(Total Active Time)** | **Fitbit Charge HR**  **(Active Minutes)** | **Jawbone UP24**  **(Active Time)** |
| --- | --- | --- | --- | --- |
| 362 ± 188 | 58 ± 40 | 818 ± 88 | 70 ± 59 | 112 ± 59 |
